# Ectopic expression of a mechanosensitive channel confers spatiotemporal resolution to ultrasound stimulations of neurons for visual restoration

**DOI:** 10.1038/s41565-023-01359-6

**Published:** 2023-04-03

**Authors:** Sara Cadoni, Charlie Demené, Ignacio Alcala, Matthieu Provansal, Diep Nguyen, Dasha Nelidova, Guillaume Labernède, Jules Lubetzki, Ruben Goulet, Emma Burban, Julie Dégardin, Manuel Simonutti, Gregory Gauvain, Fabrice Arcizet, Olivier Marre, Deniz Dalkara, Botond Roska, José Alain Sahel, Mickael Tanter, Serge Picaud

**Affiliations:** 1 Sorbonne Université, INSERM, CNRS, Institut de la Vision, Paris, France; 2 Physics for Medicine Paris, INSERM, CNRS, École Supérieure de Physique et de Chimie Industrielles (ESPCI Paris), Paris Sciences et Lettres (PSL) Research University, Paris, France; 3 Institute of Molecular and Clinical Ophthalmology Basel, Basel, Switzerland; 4 Department of Ophthalmology, The University of Pittsburgh School of Medicine, Pittsburgh, PA USA; 5 Department of Ophthalmology and Vitreo-Retinal Diseases, Fondation Ophtalmologique Rothschild, Paris, France; 6 Centre Hospitalier National d’Ophtalmologie des XV-XX, Paris, France

**Keywords:** Biotechnology, Techniques and instrumentation

## Abstract

Remote and precisely controlled activation of the brain is a fundamental challenge in the development of brain–machine interfaces for neurological treatments. Low-frequency ultrasound stimulation can be used to modulate neuronal activity deep in the brain, especially after expressing ultrasound-sensitive proteins. But so far, no study has described an ultrasound-mediated activation strategy whose spatiotemporal resolution and acoustic intensity are compatible with the mandatory needs of brain–machine interfaces, particularly for visual restoration. Here we combined the expression of large-conductance mechanosensitive ion channels with uncustomary high-frequency ultrasonic stimulation to activate retinal or cortical neurons over millisecond durations at a spatiotemporal resolution and acoustic energy deposit compatible with vision restoration. The in vivo sonogenetic activation of the visual cortex generated a behaviour associated with light perception. Our findings demonstrate that sonogenetics can deliver millisecond pattern presentations via an approach less invasive than current brain–machine interfaces for visual restoration.

## Abstract (web summary)

Sonogenetics provides neuron-specific activation at high spatiotemporal resolution ex vivo in retina and in vivo deep in the visual cortex using the AAV gene delivery of a mechanosensitive ion channel and low-intensity ultrasound stimulations.

## Main

Brain–machine interfaces (BMIs) based on multielectrode arrays (MEAs) have met with increasing success in peripheral sensory system rehabilitation strategies as well as for restoring hearing in the cochlea or sight in the retina^1,2^. The restoration of vision is the most demanding challenge for BMIs, as it ultimately requires the 13 Hz rate transmission of complex spatial patterns^3^. Although form perception can be achieved by epicortical or intracortical implants^4,5^, the lack of long-term sustainability has intensified the search for the non-contact distant activation of neuronal circuits. Optogenetic therapy has provided an alternative, as demonstrated on the retina even at the clinical level^6^. Despite encouraging animal studies^7–9^, approaches for the optical stimulation of the cortex are hindered by the dura mater and by brain scattering as well as the absorption of light requiring invasive light guides^10^.

**Fig. 1:**
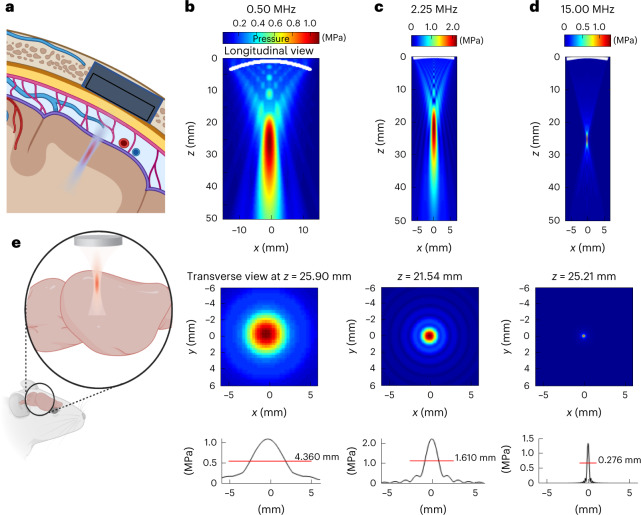
Sonogenetics using focused US beams for visual restoration through the intact dura mater: impact of ultrasonic transmission frequency. **a**, Concept of visual restoration with US matrix arrays implanted in a cranial window for the localized US neuromodulation of the primary visual cortex in humans. The US beam can be adaptively focused at different locations in the V1 cortex as it passes through the intact dura mater as well as subdural and subarachnoid spaces. **b**, Proof-of-concept setup used in this study for V1 sonogenetic activation in rodents, using a high-frequency focused transducer on a craniotomized mouse. **c**, Characterization of the radiated field for the 0.5 MHz transducer used in this study. A longitudinal view of the maximal pressure for a monochromatic acoustic field radiated at 0.5 MHz by the 25.40-mm-*Ø*, 31.75-mm-focus transducer (top). The maximum pressure is reached at 25.9 mm, slightly closer to the transducer than the geometric focal point, which is a documented effect. The transverse section of the maximum pressure field at depth *z* = 25.9 mm (middle). A one-dimensional profile of this transverse section giving the FWHM of the focal spot (4.36 mm at 0.5 MHz) (bottom). **d**, Same characterization for the 2.25 MHz 12.7-mm-*Ø* 25.4-mm-focus transducer. **e**, Same characterization for the 15 MHz 12.7-mm-*Ø* 25.4-mm-focus transducer. Note that the maximum pressure is reached very close to the geometric focus (25.21 mm versus 25.40 mm for the geometric focus) for this configuration. The FWHM of the focal spot is 0.276 mm. Panels **a** and **e** are created with Biorender.com. Source data

Ultrasound (US) waves could potentially overcome these limitations to achieve the non-contact neuromodulation of cortical and subcortical areas of the brains^11–17^. However, this neuromodulation requires a craniotomy (Fig. 1a) and the use of high US frequencies to reach the required spatial resolution. Switching from 0.5 MHz to 15.0 MHz would theoretically lead to a 30-fold improvement in resolution (Fig. 1c–e) and an ~27,000-fold improvement in neuromodulated volume. Unfortunately, most existing US neuromodulation strategies are restricted to low-frequency^15^ or mid-range^18^ transmissions resulting in poor spatial resolution (>3 mm) and/or long-lasting responses, whereas a high frequency of 30 MHz was reported to generate inhibitory neuromodulation^19^. Other attempts at high-frequency neuromodulation have resulted in higher levels of acoustic energy^20^, with risks of thermal heating^21^ and tissue damage^14^.

Sonogenetic therapy has proposed to generate neuronal mechanosensitivity by the ectopic expression of US-sensitive proteins like the TRP1 ion channel^22^, mechanosensitive ion channel of large conductance (MscL) (ref. ^23^) or auditory-sensing protein prestin^24^ using AAV gene delivery to target specific cell populations^23,25,26^, although without the spatiotemporal resolution compatible for vision restoration. A high temporal resolution was shown for MscL only in primary cultured hippocampal neurons with mutations enhancing its pressure sensitivity^27,28^—the G22S MscL mutant boosting US sensitivity of in vivo neurons^23^.

Here we have investigated if we can use the MscL channel^29^: (1) to boost the neuronal sensitivity to US not only ex vivo but also in vivo, (2) to target a locally defined subset of neurons by gene therapy, (3) to induce responses with a temporal precision (millisecond time delay and recovery) sufficient for visual restoration and (4) to gain more than one order of magnitude in spatial resolution through the in vivo use of high-frequency US at low acoustic intensities to prevent adverse effects^20^.

## Sonogenetic activation on the ex vivo retina

**Fig. 2:**
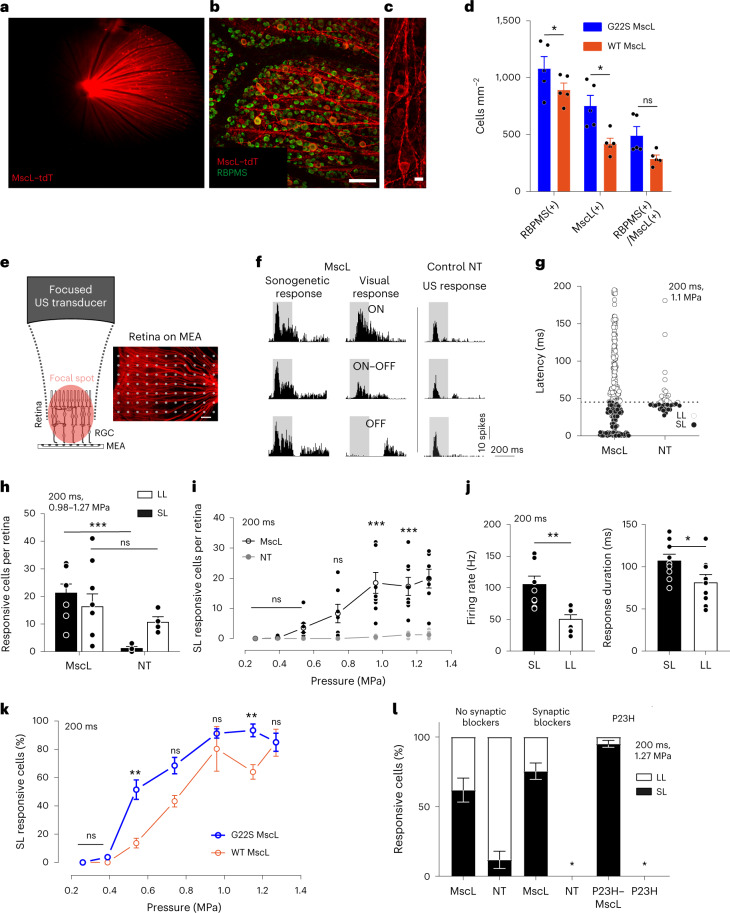
Sonogenetic therapy in rat RGCs. **a**, In vivo retinal fundus image showing MscL–tdTomato expression. **b**,**c**, Confocal stack projections across the RGC layer of a flat-mounted retina. **d**, Density of RBPMS-positive, MscL-positive and double-labelled cells (*n* = 5 WT MscL and G22S MscL retinas; *p = 0.0140, for RBPMS(+); *p = 0.0465 for RBPMS(+)/MscL(+), unpaired two-tailed *t*-test). **e**, Schematic of the experimental setup with an image of the retina on MEA electrodes. **f**, Representative peristimulus time histograms (PSTHs) for US or visual stimuli in MscL-transfected or NT RGCs (US stimuli, 15 MHz at 1.27 MPa). **g**, RGC response latencies to a 15 MHz US stimulus for MscL (*n* = 300 cells, 9 retinas) and NT retinas (*n* = 41 cells, 4 retinas). Dotted line, 45 ms latency threshold. **h**, Numbers of cells per retina responding to 15 MHz US stimuli (0.98–1.27 MPa) for MscL (*n* = 9 retinas) and NT (*n* = 4 retinas) with SL (<45 ms) or LL (>45 ms). *p = 0.0002, unpaired two-tailed *t*-test. **i**, Mean numbers of SL-responding RGCs per retina following stimulation with US stimuli of increasing pressures for MscL (*n* = 9) and NT (*n* = 4) retinas. ***p = 0.00008, ***p = 0.0010, ***p = 0.0008, multiple unpaired two-tailed *t*-tests. **j**, Maximum firing rates and response durations (SL and LL RGCs from MscL retinas in response to US stimuli of increasing pressures (0.20–1.27 MPa)) (*n* = 9 retinas, **p = 0.0017, *p = 0.0418, unpaired two-tailed *t*-test). **k**, Percentages of SL RGC cells (normalized against the maximum number of responsive cells in each experiment) responding to US stimuli for WT MscL (*n* = 3 retinas) and G22S MscL (*n* = 6 retinas) retinas. **p = 0.0065, **p = 0.0083, multiple unpaired two-tailed *t*-tests. **l**, Ratios of RGCs responding to US stimulation with SL or LL for MscL and NT retinas (*n* = 9 retinas for MscL and 4 retinas for NT), following the application of a cocktail of synaptic blockers (CNQX-CPP-LAP4, *n* = 3 retinas for both MscL and NT) and for P23H retinas with and without MscL expression (for both, *n* = 3 retinas). *Conditions with no US-elicited cell responses. Data are presented as mean values ± standard error of the mean (s.e.m.). Scale bars, 100 μm (**b**), 20 μm (**c**), 200 μm (**e**). Source data

**Extended Data Fig. 1:**
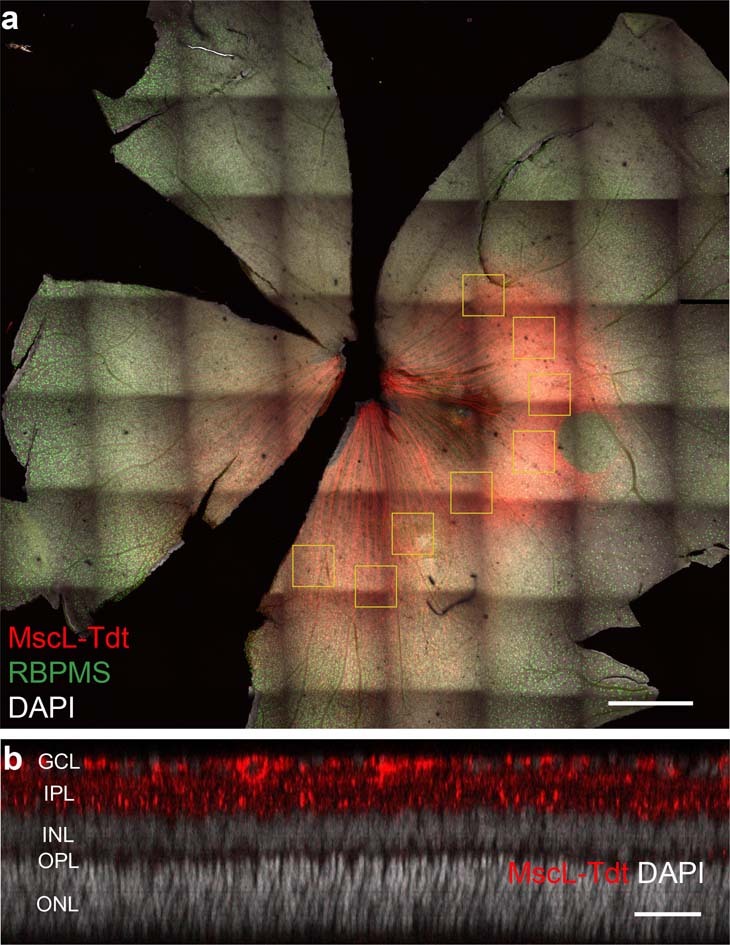
Retinal expression of MscL. (a) Whole-mount retina expressing MscL WT (red) and labeled with the RGC-specific anti-RBPMS antibody (green), with DAPI staining of the nucleus (white). Yellow boxes represent the 8 zones selected for the counting of MscL- and RBPMS-positive cells. (b) Optical section of a confocal stack showing MscL expression limited to the ganglion cell layer. The scale bars represent 1 mm in (a), 50 µm in (b). Similar results have been obtained for N = 10 retinas (5 expressing MscL WT and 5 expressing MscL G22s).

Using the retina as an easily accessible part of the central nervous system, we specifically targeted MscL into rat retinal ganglion cells (RGCs), with in vivo intravitreous delivery by an adeno-associated vector (AAV) encoding the *mscL* gene from *Escherichia coli* in its wild-type (WT) form or with the G22S mutation^28^. An AAV2.7m8 (ref. ^30^) serotype vector was used to encode MscL fused to the red fluorescent protein tdTomato, under the control of the SNCG promoter to target the RGC population^31^. On the eye fundus, tdTomato fluorescence was detected in vivo (Fig. 2a). Its expression was restricted to RGCs, as indicated by their double labelling with a specific RGC antibody, RPBMS (Fig. 2b and Extended Data Fig. 1b). The expression of the MscL channel seemed to be concentrated at the cell membrane on the soma and axon (Fig. 2c and Extended Data Fig. 1) with 24% and 46% of RPBMS-positive cells expressing tdTomato for the WT MscL and G22S MscL proteins, respectively (Fig. 2d).

**Extended Data Fig. 2:**
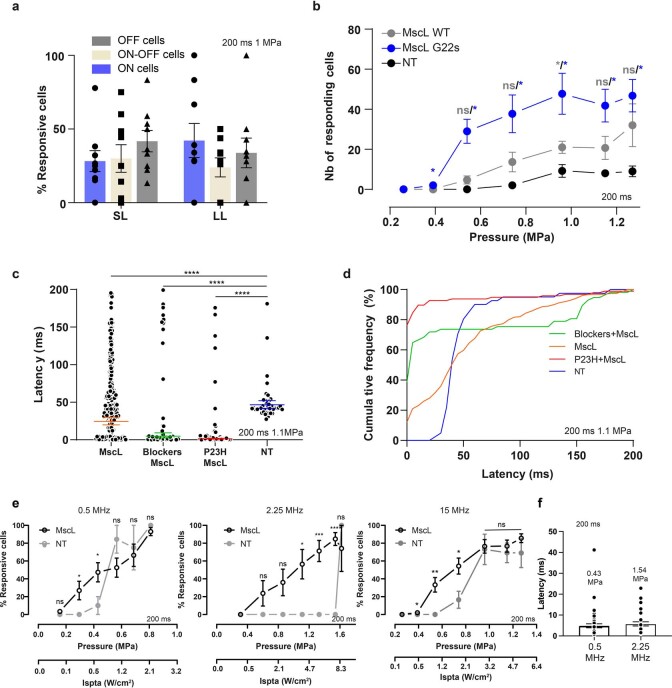
Retinal sonogenetic response characteristics for US stimuli of different frequencies. (a) Mean distributions of the different RGC cell types (ON, OFF, ON-OFF) among short (SL) and long latency (LL) responses in retinas (*n* = 9) expressing MscL (WT and G22s form) following a 15 MHz US stimulus (SD: 21.6, 28.0, 21.8 % for SL, 34.7, 19.4, 30.3 % for LL cells, for ON, ON-OFF and OFF cells respectively). (b) Mean numbers of RGCs responding to a 15 MHz stimulus of increasing acoustic pressure for MscL WT (*n* = 3), MscL G22s (*n* = 5) and NT (*n* = 4) retinas (0.39 MPa: * *p* = 0.0163; 0.54 MPa: ns *p* = 0.1480, * *p* = 0.0168; 0.74 MPa: ns *p* = 0.1334, * *p* = 0.0312; 0.96 MPa: * *p* = 0.0462, * *p* = 0.0279; 1.15 MPa: ns *p* = 0.1617, * *p* = 0.0145; 1.27 MPa: ns *p* = 0.1580, * *p* = 0.0144; unpaired two-tailed *t* test between MscL WT and NT in gray and MscL-G22s and NT in blue). (c) Scatter plots and geometric means of RGC latencies in response to a 15 MHz US stimulus for MscL (*n* = 300 cells SD: 48.8), Blockers+MscL (*n* = 57 cells, SD: 68.0), P23H + MscL (*n* = 97 cells, SD: 37.5), and NT (*n* = 41 cells, SD: 27.4) retinas (****, p = 7.3*10^-8^ for MscL and Blockers MscL vs NT and *p* < .1*10^-15^ for P23H MscL vs NT, unpaired two-tailed *t-*test on log-transformed values). (d) Cumulative frequency distributions of RGC latencies for MscL, Blockers+MscL, P23H + MscL, and NT retinas. (e) Mean percentages of cells responding to US stimuli (normalized against the maximum number of responsive cells in the experiment) of increasing acoustic pressure for 0.5 MHz (ns *p* = 0.1661;* *p* = 0.0292; * *p* = 0.0260; ns *p* = 0.8628; ns *p* = 0.1316; ns *p* = 0.7731; unpaired *t* test,), 2.25 MHz (ns *p* = 0.1474; ns *p* = 0.0522; * *p* = 0.0140; *** *p* = 0.0005; **** *p* < 0.00002; ns *p* = 0.5000; unpaired *t* test) and 15 MHz US (* *p* = 0.0382;** *p* = 0.0065; * *p* = 0.0218; ns *p* = 0.8628; ns *p* = 0.5859; ns *p* = 0.4223; unpaired *t* test) US. The lower *x* axis represents the corresponding acoustic intensity (Ispta). (f) Mean response latencies of SL cells for 0.5 and 2.25 MHz (*n* = 9 and 8 retinas). Data are presented as mean values + /- SEM. Source data

During the ex vivo recordings of the MscL-expressing retina (Fig. 2e), RGCs displayed strong and sustained ON spiking responses to focused 15 MHz US stimulation (Fig. 2f (left)) irrespective of their ON or OFF responses to light (Extended Data Fig. 2a). Many RGCs presented responses with very short latencies (SLs), namely, 12.2 ± 2.5 ms (Fig. 2f (left)), but some had long latencies (LLs) (Fig. 2g). By contrast, non-transfected (NT) retina displayed only LL responses, that is, 50.4 ± 4.2 ms (Fig. 2f (right) and Fig. 2g). Synaptic blockers (CNQX-LAP4-CPP) abolished US responses in NT retinas but not in MscL-transfected retinas, in which they decreased the number of LL US responses (LL denotes latency of more than 45 ms; Fig. 2l and Extended Data Fig. 2c,d). This observation suggests that responses in NT retinas originate upstream from RGCs, as previously reported^32^. This conclusion was supported by the absence of US response in the retinas of NT blind P23H rats having lost photoreceptors whereas transfected P23H showed a majority of SL responses (<45 ms) (Fig. 2l and Extended Data Fig. 2c,d). The geometric-mean latencies in MscL-tested groups were very different from those for the NT retina, especially in the blind P23H retina (Extended Data Fig. 2c), but the cumulative distribution of latencies further highlighted these differences (Extended Data Fig. 2d). These results suggested a natural mechanosensitivity in photoreceptors highly reminiscent of that of auditive cells in agreement with the expression of Usher proteins in both sensory cells. These proteins are known for generating the auditory mechanotransduction and probably the phototropism of photoreceptors underlying the Stiles Crawford effect^33^.

**Fig. 3:**
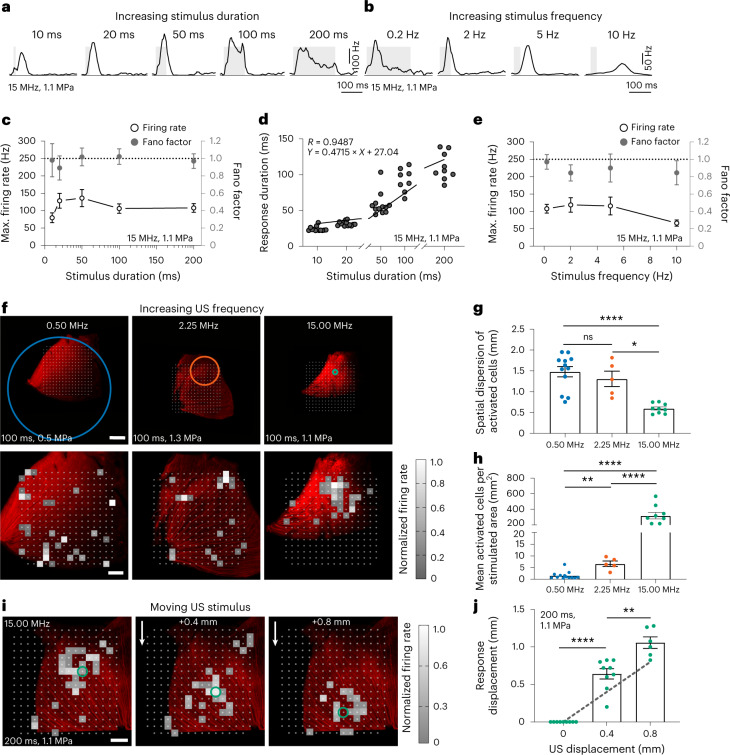
Spatiotemporal properties of sonogenetic retinal responses. **a**,**b**, Spike density functions of two RGCs from the MscL retina for 15 MHz stimulus durations and repetition frequencies (0.5 Hz repetition rate (**a**); 10, 20, 50 and 200 ms durations (**b**)). **c**, Maximum firing rates for different 15 MHz stimulus durations and mean Fano factor values for all the cells (10–20 ms, *n* = 8 retinas; 50–200 ms, *n* = 9 retinas). **d**, Correlation between response duration and stimulus duration (*n* = 9 retinas). **e**, Maximum firing rates for different stimulus repetition frequencies and mean Fano factor values for all the cells (0.2–2.0 Hz, *n* = 9 retinas; 5.0–10.0 Hz, *n* = 8 retinas). **f**, Retinas on an MEA chip and the corresponding size of the incident US pressure beam (the circles represent the FWHM and are centred on the estimated centre of the response) for 0.50, 2.25 and 15.00 MHz (top). The corresponding activation maps representing the normalized firing rates of the cells following US stimulation (bottom). Each square box represents an electrode with at least one US-activated cell. **g**,**h**, Spatial dispersions of activated cells (**g**) and ratios of the number of activated cells to the stimulated area for the three US frequencies (**h**); ****p = 0.00002 (**g**), *p* = 0.00006 (15.00 versus 2.25 MHz) and *p* = 0.00005 (15.00 versus 0.50 MHz) (**h**); **p = 0.0008, *p = 0.0169, unpaired two-tailed *t*-test. Here *n* = 12 retinas for 0.50 MHz (0.29–0.68 MPa), *n* = 5 retinas for 2.25 MHz (1.11–1.62 MPa) and *n* = 9 retinas for 15.00 MHz (1.12–1.27 MPa). **i**, Heat maps showing activated cells in the MscL retina following displacements (0.4 and 0.8 mm) of the US transducer. The circles represent the estimated centre of the response. **j**, Relative displacements of the centre of the response following the displacement of the 15 MHz US transducer. ****p = 0.00001, **p = 0.0018, unpaired two-tailed *t*-test. Here *n* = 9, 9 and 6 positions for 4, 4 and 2 retinas for displacements of 0, 0.40 ± 0.20 and 0.80 ± 0.18 mm (s.d.), respectively. The grey dotted line represents the theoretical displacement. Data are presented as mean values ± s.e.m. Scale bars, 1.0 mm (**f**, top); 0.5 mm (**f** (bottom) and **i**). Source data

MscL expression decreased latency and increased the mean number of cells per retina responding to US (Fig. 2h). SL-responding cells expressing MscL were sensitive at much lower US pressures than NT cells and their number increased with the US pressure (Fig. 2i). SL US responses also involved higher firing rates and were more sustained than LL US responses (Fig. 2j). Moreover, we observed that the G22S mutation further enhanced the sensitivity of SL RGCs to lower US pressures (Fig. 2k and Extended Data Fig. 1b). We subsequently restricted our analyses to SL US responses (<45 ms). Neurons responded even to very short stimulation durations (10 ms), with responses showing a fast return to the control level of activity (Fig. 3a). US response durations were correlated with the stimulus duration, although a reduction in the firing rate occurred for longer stimuli (>100 ms) (Fig. 3c,d). Using different stimulus repetition rates, RGCs were able to follow rhythms up to a 10 Hz frequency (Fig. 3b–e). The Fano factor indicated that the response had low variability in the spike count and possibly high information content (Fig. 3c–e).

**Extended Data Fig. 3:**
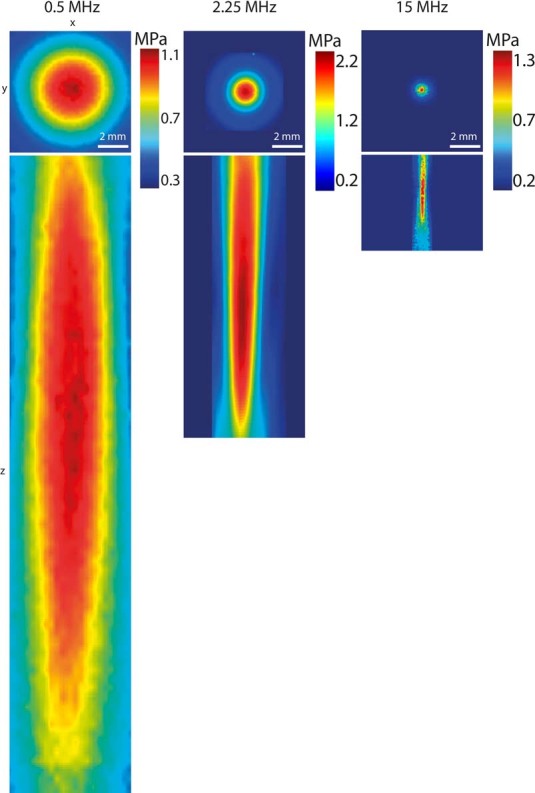
Experimentally measured US pressure fields. US pressure fields near the focus for 0.5, 2.25 and 15 MHz focused transducers, measured in water. Color-coded pressure maps in the *xy* and *xz* planes, for 0.5, 2.25 and 15 MHz.

We then investigated whether different US frequencies (0.50, 2.25 and 15.00 MHz) affected the spatial resolution of the response, in accordance with the measured US pressure fields (Extended Data Fig. 3). Transducers were designed with a similar focal distance and numerical aperture, for the transmission of focused beams over different frequency ranges (0.50, 2.25 and 15.00 MHz, corresponding to wavelengths of 3.0, 0.7 and 0.1 mm, respectively) (Fig. 1c–e). Features of responses evoked by the different US frequencies were found to be similar (Extended Data Fig. 2e,f), although increasing the frequency from 0.5 MHz (typical of neuromodulation) (Fig. 1c) to 15.0 MHz (Fig. 1e) reduced the focal spot by a factor of ~4,100 with our transducers. Cells responding to US were widespread over the recorded area for 0.50 and 2.25 MHz, but appeared to be more confined for 15.00 MHz (Fig. 3f), despite similar acoustic parameters (100 ms at 1.1 and 1.3 MPa) for the 2.25 MHz and 15.00 MHz beams. The acoustic pressure at 0.5 MHz was lower (0.5 MPa) due to electric-power limitation of our electronics. The spatial dispersion of activated cells decreased significantly from 1.48 ± 0.12 mm and 1.30 ± 0.18 mm at 0.50 MHz and 2.25 MHz, respectively, to 0.59 ± 0.03 mm at 15.00 MHz (Fig. 3g). This spatial dispersion was consistent with the size of the measured US pressure fields (Fig. 1c–e); for the 0.50 MHz transducer, the focal spot was much larger than the MEA chip. The density of activated cells increased significantly with increasing US frequency but on a smaller area (Fig. 3h). US stimulation is more effective at higher frequencies, because lower acoustic power values are required to activate an equivalent number of cells. Indeed, even if the acoustic intensities at 2.25 and 15.00 MHz were fairly similar, the acoustic power delivered was almost two orders of magnitude lower at 15.00 MHz (0.03 W) than at 2.25 MHz (0.82 W). At 15.00 MHz, moving the focal spot of the US probe above the retina triggered a shift in the area of responding cells (Fig. 3i). The response centre was found to move in accordance with the displacement of the US transducer (Fig. 3j). These results demonstrate that our sonogenetic therapy approach can efficiently activate neurons with a millisecond and submillimetre precision.

## Spatiotemporal resolution in vivo on the visual cortex

**Fig. 4:**
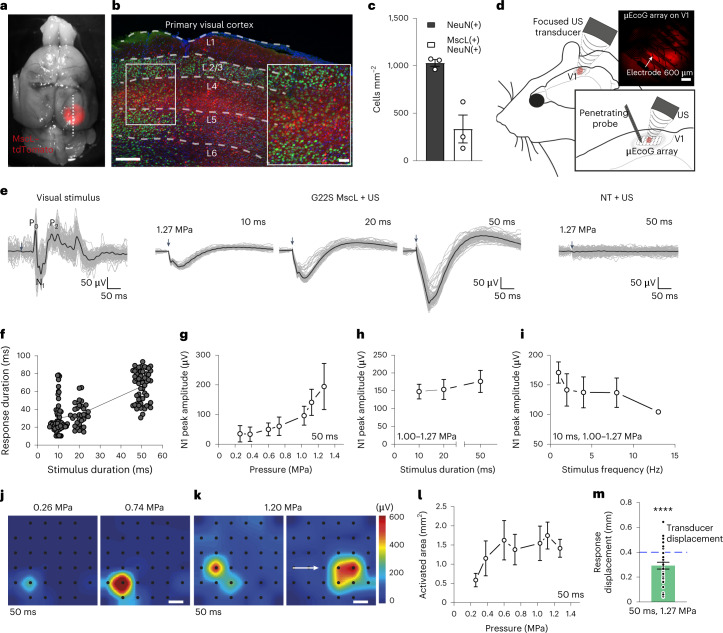
Spatial resolution of in vivo sonogenetic therapy in V1 cortical neurons. **a**, Image of a rat brain expressing G22S MscL–tdTomato (red) in V1. **b**, Confocal stack projection of a sagittal brain slice expressing G22S MscL–tdTomato (red) and labelled with anti-NeuN antibody (green) and DAPI (blue). The layers of V1 are delineated by the dashed white lines. Magnification of layer 4 of V1 (lower right). **c**, Densities of NeuN-positive, MscL-positive and double-labelled cells for three brain slices. **d**, Schematic of the setup used for in vivo electrophysiological recordings and US stimulation. µEcoG electrode array placed on V1 of an MscL-transfected rat (top right). **e**, Visual-evoked cortical potentials in response to a 100 ms flash (left). Sonogenetic-evoked potentials for 15 MHz US stimuli of various durations (middle). Absence of US responses in an NT rat to a 15 MHz stimulus (right). The black traces represent the mean evoked potentials over 100 trials, individually illustrated by the grey traces. The black arrows indicate the stimulus onsets. **f**, Duration of sonogenetic µEcog responses for the stimuli of different durations (10 ms, *n* = 58 trials; 20 ms, *n* = 32 trials; 50 ms, *n* = 56 trials on 6 animals). **g**–**i**, N1 peak amplitudes for increasing US pressure (**g**), increasing duration (**h**) and increasing frequency (**i**) (*n* = 6 rats). **j**,**k**, Pseudocolour activation maps for the stimuli of increasing US pressure (**j**) and for a horizontal displacement of the US transducer by 0.8 mm (**k**) (the arrow indicates the direction of displacement). Each black dot represents an electrode of the array. The colour bar represents the N1 peak amplitude in microvolts. **l**, Mean activated areas for various US pressure values (*n* = 6 animals). **m**, Relative displacement of the activation centre to the previous position following movement of the US transducer by 0.4 mm. Here *p* = 1 × 10^–12^, one-sample two-tailed *t*-test, *n* = 37 positions on 6 animals (mean, 0.29 ± 0.16 mm (s.d.)). Data are presented as mean values ± s.e.m. Scale bars, 200 and 50 μm (**b**); 300 μm (**j** and **k**). Source data

We investigated whether this approach could also be applied to the brain in vivo through a cranial window (Fig. 1a,b). As the G22S mutation enhanced the US sensitivity of RGCs ex vivo, we expressed G22S MscL in cortical neurons of the primary visual cortex (V1) in rats. We injected AAV9.7m8 encoding the G22S MscL channel fused to tdTomato under the control of the neuron-specific CamKII promoter into V1. The tdTomato fluorescence was detected in the brain (Fig. 4a) and in cortical slices, particularly in layer 4 (Fig. 4b). Staining with an anti-NeuN antibody showed that 33.4% of cortical neurons in the transfected area expressed tdTomato (Fig. 4c).

To measure the responses to 15 MHz US stimulations, we placed a micro-electrocorticography (µEcoG) electrode array on the cortical surface of V1 (Fig. 4d). In NT animals, no US-evoked signal was recorded (Fig. 4e (right), *n* = 3 rats), whereas in V1 expressing G22S MscL, the US stimulation of the cortical surface elicited large negative µEcoG potentials (Fig. 4e (middle), *n* = 6 rats). These US-evoked negative deflections were different from the recorded visual-evoked potentials (Fig. 4e (left)). Amplitudes and durations of the US responses were clearly related to the duration of US stimulations (Fig. 4f,h) and US pressures (Fig. 4g). V1 cortical responses were again able to follow a repetition rate of up to 13 Hz (Fig. 4i) even if the peak amplitude slightly decreased for increasing stimulation frequencies.

The peak depolarization of each channel was measured and linearly interpolated to build pseudocolour activation maps showing sizes of the US-responding cortical area dependent on the US pressure from 0.26 MPa (0.58 ± 0.17 mm^2^, *n* = 6 rats) to 1.27 MPa (1.41 ± 0.23 mm^2^, *n* = 5 rats) (Fig. 4j–l). When the US probe was moved laterally, the source of the generated neuronal activity moved in a similar direction (Fig. 4k). The spatial location of the evoked potentials moved by 0.29 mm (±0.09 mm, *n* = 6 rats) from the previous location (Fig. 4m and Extended Data Fig. 5), even though we moved the US transducer in 0.40 mm steps. This discrepancy between the displacement of the activated area and movement of the transducer was certainly related to the 0.3 mm discrete spatial pitch distribution of the electrodes and the lateral spread of activity in the circuit. These results suggest that our approach to sonogenetic therapy could yield a spatial resolution of within 400 µm for stimulations at 15 MHz, the focal spot of our 15 MHz transducer being 276 µm wide (Fig. 1d). This opens up the possibility of targeting small areas (down to 0.58 mm^2^ for 0.26 MPa), depending on the pressure level. These very localized US-evoked responses, their dependence on the position of the US probe and their SLs confirmed that they were due to the activation of G22S MscL-expressing neurons and not to an indirect response related to auditory activation, as suggested previously^34,35^.

**Fig. 5:**
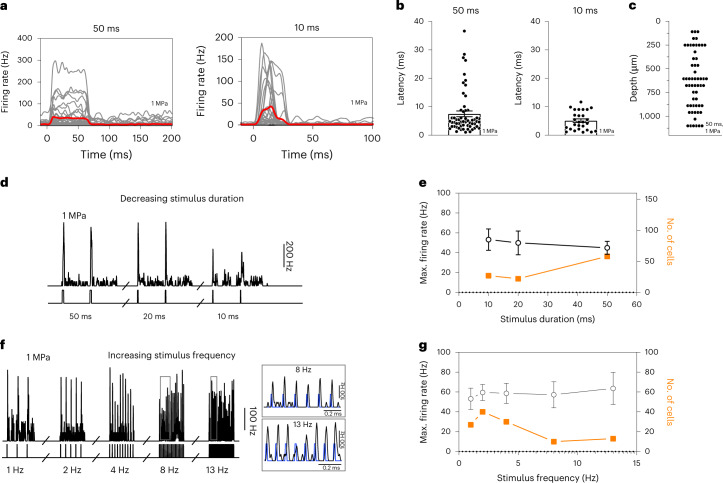
Temporal resolution of in vivo sonogenetic cortical activation. **a**, Spike density functions (SDF) of 58 and 27 neurons recorded with a penetrating MEA in MscL-transfected rats following US stimulation for 50 and 10 ms (red, mean trace; grey, individual cells). **b**, Response latencies following 50 and 10 ms of US stimuli (50 ms, *n* = 58 cells, mean of 7.5 ± 7.6 ms (s.d.), 7 rats; 10 ms, *n* = 27 cells, mean of 5.1 ± 3.2 ms (s.d.), 5 rats). **c**, Depth of US-responding cells (*n* = 58) in MscL-expressing rats (*n* = 7). **d**, Instantaneous SDF of responses to US stimuli of different durations (1 Hz stimulus repetition frequency). **e**, Maximum firing rates (*n* = 27, 22 and 58 cells; s.d., 55.8, 56.2 and 49.8 ms for 10, 20 and 50 ms stimulation, respectively) and numbers of activated neurons on US stimulations of different durations (US pressure, 1 MPa). **f**, Instantaneous SDF of responses to US stimuli of different repetition frequencies (10 ms stimulus duration). **g**, Mean maximum firing rate and number of activated neurons on US stimulation at different stimulus repetition frequencies (10 ms, 1 MPa, *n* = 27, 40, 30, 10, 13 cells; s.d., 55.8, 50.8, 55.7, 41.5, 58.2 Hz). Data are presented as mean values ± s.e.m. Source data

**Extended Data Fig. 4:**
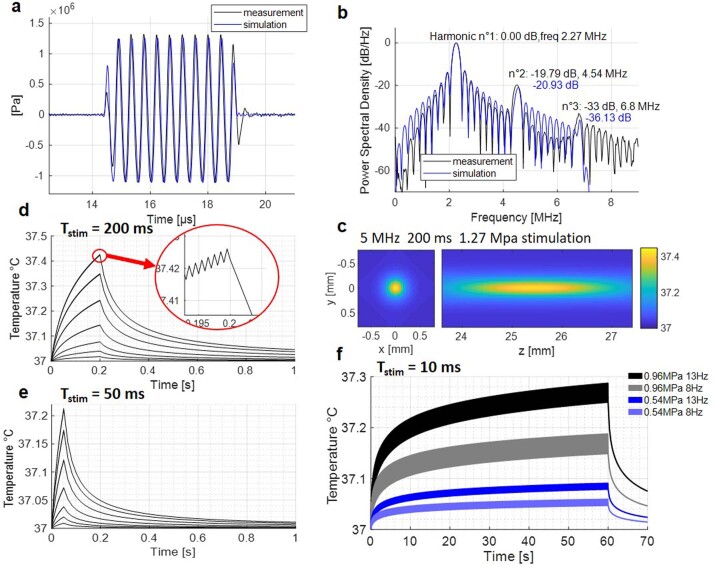
Simulated acoustic fields and temperature increases. (a) Comparison between a water tank measurement at the focus with a calibrated hydrophone (black) obtained with the 2.25 MHz transducer and reaching -1.11 MPa peak negative pressure, and a simulated waveform at the focus (blue) reaching the same negative pressure. The two waveforms match very well (0.42% error) ensuring a good match between our simulation setup and physical parameters. (b) Power spectral density of the measured (black) and simulated (blue) waveforms, showing that simulations can be used to estimate the importance of non-linear propagation. A second harmonic 20 dB below the fundamental indicates a factor of 100 in terms of energy, meaning that absorption can be calculated in a linear approximation. (c-f) Thermal simulations are performed in a two-fold process corresponding to a worst-case scenario (see methods): propagation in a water medium, and thermal absorption in a brain-mimicking medium. (h) 3D temperature map at the end of a 200 ms stimulation (at 15 MHz and 1.27 MPa). (d) Temperature rise at the focus for a 15 MHz 200 ms stimulation with the 7 pressures used in Fig. 1I (0.26, 0.39, 0.54, 0.74, 0.96, 1.15, 1.27 MPa). A zoom on the increasing curve reveals the fluctuations due to the 1 kHz on-off cycles. (e) Temperature rise at the focus for a 15 MHz 50 ms stimulation with the same 7 pressures. (f) Temperature rise at the focus for 15 MHz 10 ms stimulations (1 kHz modulation) at a repetition rate of 8 Hz and 13 Hz (used in Fig. 3o), for focus pressures of 0.96 MPa and 0.54 MPa. Source data

**Extended Data Fig. 5:**
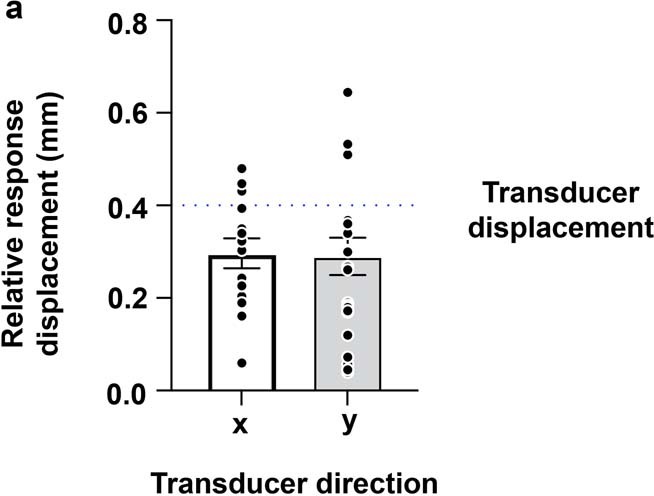
In vivo response displacement to US stimulation. (a) Relative displacement of the activation center to the previous position following movement of the US transducer by 0.4 mm in the x and y direction (*n* = 37 positions on 6 animals). Data are presented as mean values + /- SEM. Source data

When recording with penetrating electrode arrays (Fig. 4d), V1 neurons expressing G22S MscL generated sustained responses even to 10-ms-long 15 MHz US stimuli (Fig. 5a) with latencies shorter than 10 ms (5.10 ± 0.62 ms, *n* = 27 cells) (Fig. 5b), consistent with direct US activation. Responding neurons were recorded at various cortical depths, ranging from 100 µm to 1.00 mm (Fig. 5c), the focal spot diameter of the US probe being 3.75 mm in the *x*–*z* plane. Deep neurons reliably responded to the stimuli of decreasing duration, from 50 ms to 10 ms, with similar firing rates, whereas longer stimuli induced responses in a broader population of neurons (Fig. 5d–e). To investigate if a US pattern could be applied for visual restoration at a refreshing rate of up to 13 Hz, we progressively increased the sequence of stimuli. Cortical neurons were able to generate distinct responses to each US stimulus up to a 13 Hz repetition rate (Fig. 5f), but the number of responding cells decreased with increasing stimulus frequency (Fig. 5g). No major tissue temperature increase is expected even at this stimulation rate (Extended Data Fig. 4).

## Behavioural response to the sonogenetic stimulation of the visual cortex

**Fig. 6:**
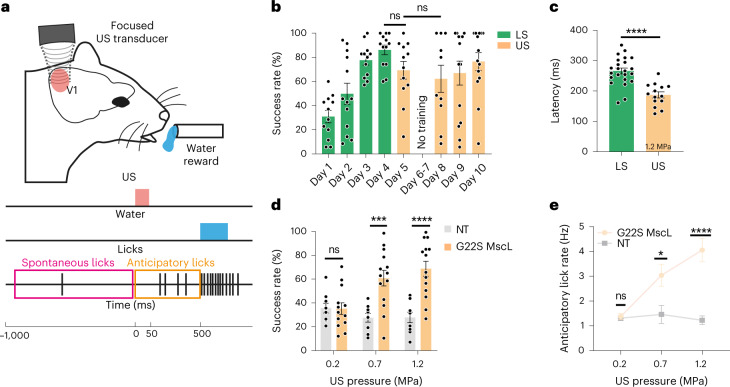
Behavioural response induced by the sonogenetic activation of the V1 cortex in mice following associative visual training. **a**, Schematic of the behavioural task performed by mice. Water-restricted animals trained in an associative learning paradigm for light stimulation (LS) with a water reward are subjected to either an LS of the eye (days 1–4) or the US stimulation of V1 at 15 MHz (days 5 and 8–10). **b**, Mean rates of successful trials for 4 days of training during the learning of association between LS (green, 50 ms) and water reward followed by US stimulation (orange, 1.2 MPa) for G22S MscL-transfected mice (between day 4 of LS and day 5 of US; 50 ms at 1.2 MPa; ns, *p* = 0.0570). Between day 5 of US and day 8 of US, 50 ms at 1.2 MPa; ns, *p* = 0.6079, two-tailed unpaired *t*-test; mean, 30.9%, 49.9%, 77.6%, 86.2%, 69.3%, 62.3%, 66.9%, 76.5%; s.d., 17.9%, 31.2%, 13.9%, 14.1%, 25.4%, 35.4%, 37.1%, 27.7%; *n* = 14 animals. **c**, Mean times to first lick after light (50 ms) and US stimulation (50 ms at 1.2 MPa) (****p = 0.0000290, two-tailed unpaired *t*-test, *n* = 23 and *n* = 14 animals; mean, 265.9, 187.1 ms and s.d., 46.5, 37.3 ms for LS and US, respectively). **d**, Mean rates of successful trials over 4 days of US stimulation for NT and G22S MscL-transfected mice, following 50 ms of US stimulation at increasing US pressures (ns *p* = 0.9452, ***p = 0.0003, ****p = 0.0000296, two-tailed unpaired *t*-test, for 0.2, 0.7 and 1.2 MPa, respectively; *n* = 14 animals; mean, 35.2%, 60.8%, 68.7% and s.d., 17.5%, 24.4%, 23.6% for G22S MscL; *n* = 9 animals; mean, 35.7%, 27.5%, 27.8% and s.d., 12.4%, 11.0%, 13.2% for NT). **e**, Session anticipatory lick rates for NT and G22S MscL-transfected mice at increasing US pressures (ns *p* = 0.6934, *p = 0.0119, ****p = 0.0000340, two-tailed unpaired *t*-test, for 0.2, 0.7 and 1.2 MPa, respectively; *n* = 14 animals; mean: 1.4, 3.0, 4.1 and s.d., 0.4, 1.7, 1.8 Hz for G22S MscL and *n* = 9 animals; mean, 1.3, 1.4, 1.2 and s.d., 0.3, 1.1, 0.5 Hz for NT). Data are presented as mean values ± s.e.m. Source data

**Extended Data Fig. 6:**
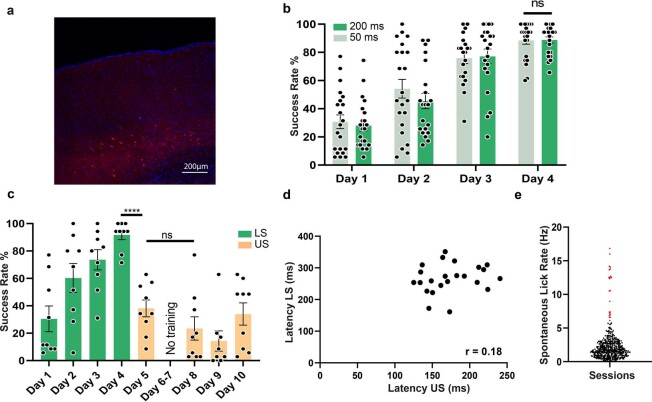
MscL G22S expression with the US and light-associative training in mice. (a) Confocal stack projection of a sagittal brain slice expressing MscL G22s-tdTomato (red) and labeled with DAPI (blue). Similar results have been obtained on N = 3 animals. (b) Head-fixed and water-restricted mice were trained for four days to respond to a full-field stimulation of one eye (200 and 50 ms) that preceded a water reward. Mice responded by licking before (anticipation — successful trial) or after the delivery of water (failure). The mean success rate increased progressively and mice learned the task (upon 50 ms and 200 ms light stimulation) after four days of training (ns *p* = 0.9387, two-tailed unpaired *t* test, Mean: 27.9, 45.4, 77.1, 88.8, SD: 17.4, 24.8, 23.6, 10.4% for 200 ms, Mean: 30.7, 54.2, 75.9, 88.5, SD: 22.2, 31.0, 17.5, 12.8% for 50 ms). (c) Mean rates of successful trials in non-transfected (NT) mice for 4 days of training with light stimulation (50 ms, LS green) and for 4 days of US stimulation (US orange) (Between Day 4 LS and Day 5 US: 50 ms 1.2 MPa, ****, *p* = 0.0000047, two-tailed unpaired *t* test. Between Day 5 US and Day 8 US: 50 ms 1.2 MPa, ns, *p* = 0.1850. Mean: 30.5, 60.3, 73.6, 91.7, 38.1, 23.5, 14.3, 34.0, SD: 28.2, 31.6, 22.1, 10.3, 18.5, 25.5, 21.1, 24.4 %). (d) Pearson correlation scatter plot for time to first lick after either light (LS) or US stimulation. (e) Identification and exclusion of outlier sessions (in red) based on the ROUT method, (Q = 1%) for the session spontaneous lick rate measured on a 1 s time window prior to all trials of the session e Q_1_ = 0.9 Hz, Median = 1.7 Hz, Q_3_ = 2.8 Hz, Mean= 2.3 Hz, SD = 2.3 Hz. Data are presented as mean values + /- SEM. Source data

To define if the US-elicited synchronous activation of MscL-expressing excitatory cortical neurons can induce light perception, we assessed the mouse behaviour during an associative learning test including 15 MHz US stimulation of V1 in G22S MscL-transfected (*n* = 14) and NT (*n* = 9) animals (Fig. 6 and Extended Data Fig. 6). Mice subjected to water deprivation were trained to associate the visible-light stimulation of one eye with a water reward (Fig. 6a)^36^. This task was learned within four days, as indicated by the increasing success rate during this period, from 30.9 ± 17.9% (standard deviation (s.d.)) to 86.2 ± 14.1% (s.d.) for G22S MscL-transfected mice (Fig. 6b). The success rate was determined by assessing the occurrence of an anticipatory lick between the light onset and the release of water reward 500 ms later (Fig. 6a). Only mice reaching a 60% success rate on the fourth day were retained for this analysis, and sessions showing a compulsive licking rate were excluded. Following cortical US stimulation on day 5, G22S MscL-transfected mice achieved a success rate of 69.3 ± 25.4% (s.d.), the difference of which showed no statistical difference with the success rate following light stimulation (LS) on day 4 (Fig. 6b). After a pause during the weekend (days 6–7), the animals retained the task, their success rates showing no statistically significant differences with the one following LS (Fig. 6b). By contrast, in NT animals, the success rate following the US stimulation of their visual cortex dropped to 38.1 ± 18.5% (s.d.), and the difference with the success rate following LS on the fourth day was highly significant (*p* < 0.0001) (Fig. 6d and Extended Data Fig. 6). In the AAV-injected mice, we found that the latency of the first anticipatory lick was shorter for sonogenetic stimulation (187.1 ± 37.3 ms; *n* = 14 (s.d.)) than for stimulation with a light flash (265.9 ± 46.5 ms; *n* = 23 (s.d.)) (Fig. 6c and Extended Data Fig. 6d). This SL for the US response is consistent with the faster activation of cortical neurons for sonogenetic stimulation than for LS of the eye (Fig. 4e). In transfected mice, success rates increased with pressure (Fig. 6d), suggesting a brighter and/or a larger US-elicited percept with a greater US pressure, as described with increasing currents in human patients^4^. Interestingly, the licking frequency during 500 ms before delivery of the water reward also increased with US pressure (Fig. 6e). These results suggest that the sonogenetic stimulation of the visual cortex generates a perception in mice that is probably associated with a visual perception, although more complex visual behaviours (as form discrimination) would be required for a demonstration.

## Safety issues

Our sonogenetic approach greatly decreased the US pressure required for the activation of RGCs and V1 cortical neurons with stimulation sequences remaining below FDA safety limits (510(k), Track 3) for US imaging (for example, for a 10 ms US stimulus of 0.6 MPa, the non-derated spatial peak temporal peak intensity (Isptp) is 12.00 W cm^–2^ and the non-derated Ispta value is 0.12 W cm^–2^). These very low acoustic pressures and acoustic intensities prevent tissue damage, as they are similar to those that have been widely used in clinical diagnostic imaging for decades^37^. Moreover, the simulations of US-induced heating in brain tissue revealed that typical US parameters (that is, 20 ms at 1.27 MPa) (Fig. 4e–h) increased the local temperature by an estimated 0.12 °C, with even high repetition rates (up to 13 Hz), leading to a moderate temperature increase (<0.30 °C) (Extended Data Fig. 4c–f). These low temperature fluctuations (corresponding to ‘worst-case’ scenarios as we used non-derated US parameters) and stimulation sequences compliant with FDA limits suggest that our approach had no toxic side effects and that US-elicited responses were not temperature driven and were therefore probably mediated by the mechanical activation of MscL channels by US. The fact that acoustic intensities and pressures used here remained far below the FDA requirements for conventional ultrasonic imaging in clinics (https://www.fda.gov/media/71100/download) and generated very low temperature increase in comparison with thermal damaging effects^38^ raises high hopes for a smooth clinical translation. Moreover, a very recent safety study^19^ demonstrated an absence of brain tissue damage using high-frequency activation at ten times higher acoustic intensities (continuous insonication at 11.80 W cm^–2^ compared with our worst-case spatial peak temporal average intensity (Ispta) of 1.56 W cm^–2^ for repeated stimulations at 13 Hz rate).

## Conclusions

The development of remotely controlled cortical and subcortical deep neuronal stimulation techniques is of considerable interest for the treatment of diverse neurological diseases and sensory handicaps. Most previous sonogenetic studies focused on the use of low-frequency US^22–24^ as in the recent demonstration of MscL-based sonogenetic activation in mouse brain^23^. However, such low-frequency US waves lead to limited centimetre spatial resolutions (∼5 × 5 × 45 mm^3^) and an uncontrolled spatial-beam distribution. An alternative approach to spatially containing US stimulations involves the use of higher US frequencies, but this was thought to demand higher energy levels, exceeding safety limits and favouring tissue damage^20^. The bacterial MscL channel has been reported to sensitize neurons to US^23,27,28^ and to lower the pressure for neuronal activation, but its use for high-spatiotemporal-resolution sonogenetic stimulation has yet to be shown to be effective in vivo. We here showed that that US activation of G22S MscL expressed in retinal or cortical neurons resulted in responses with millisecond latencies and a spatial resolution of at least 400 µm in the *x*–*y* plane at 15 MHz frequency. The subsequent neuronal activation throughout the depth of the visual cortex (Fig. 5n–p) led to a behavioural motor response, suggesting light perception by the animal. These sonogenetic responses were genuinely related to MscL expression, as they were not observed in NT animals. Following previous demonstrations that the MscL channel is a suitable sonogenetic actuator^23,27,28^, we provide further evidence that the MscL channel has appropriate kinetics for the activation of neurons at a precise spatiotemporal resolution both in situ and in vivo.

The temporal precision of sonogenetics is lower than that achieved with optogenetics (>40 Hz) by the fastest opsins^39^ and ChrimsonR (ref. ^40^), which can successfully restore vision at the retinal level in patients^6^. MscL only follows a 13 Hz frequency in vivo, which is in the same range as the 5–20 Hz achieved in vivo by the very sensitive opsin, ChRmine (ref. ^41^), a frequency range probably sufficient for vision^3^. The discovery of ChRmine has enabled investigators to stimulate deep into the rodent brain even from above the skull^41^. Future studies will have to examine the spatial resolution of this approach and how it compares to sonogenetics. As for all the gene therapies in non-dividing cells, both optogenetic and sonogenetic therapies are expected to be lifelong lasting as indicated by gene therapy in congenital Leber congenital amaurosis, although it did not stop the ongoing degeneration of photoreceptors in patients^32^.

Restoration of form vision at the cortical level was previously achieved with 0.5 to 1.0 mm surface electrodes spaced more than 1.0 mm apart^5^ or with 1.5-mm-long penetrating electrodes spaced 400 µm apart^4^. The spatial resolution of the proposed sonogenetic therapy, therefore, appears to be compatible with the cortical restoration of form vision but with a remote non-contact device. To preserve this spatiotemporal resolution, the US stimulator will require to be placed directly above the dura mater or above a US transparent artificial skull^42^. At 15 MHz, the typical penetration depth with negligible heating is 20 mm. Moreover, the resolution of the approach could be increased by using gene therapy to drive the expression in specific cell populations and cell compartments^31,43^. Further studies are required to generate an interface for coding visual information into US patterns transmitted by an ultrasonic matrix array onto the visual cortex at a video rate. To reduce the US load, visual restoration can take advantage of an event-based camera, heat-sensitive camera or depth-filtering imaging to limit the active pixel numbers in an image^44–46^. Therefore, our approach provides great hope for the development of high-resolution visual restoration at the cortical level, through its unique combination of a rapid response, high spatial resolution and cell selectivity with promoters. Even if this approach requires craniotomy, as for other existing visual prostheses, it provides a less invasive approach based on deep and distant cortical activation from above the dura mater following AAV cortical injections. More generally, it paves the way for a new type of genetic-based BMI capable of compensating for disabilities and suitable for use in treatments of neurological disorders.

## Methods

### Animals

Experiments were conducted in accordance with the National Institutes of Health Guide for the Care and Use of Laboratory Animals. Protocols were approved by the Local Animal Ethics Committee (Committee Charles Darwin no. 5, registration nos. 9529 and 26889) and conducted in agreement with Directive 2010/63/EU of the European Parliament. Long–Evans male rats aged between 2 and 12 months and WT male mice (C57BL/6J) aged 9 weeks were obtained from Janvier Laboratories; P23H (line 1) male transgenic rats (9–22 months) were raised locally.

### Plasmid cloning and AAV production

Plasmids containing the *E. coli mscL* sequence in the WT form and with the G22S mutation were obtained from Francesco Difato (Addgene plasmids #107454 and #107455)^28^. To target RGCs, the SNCG promoter^31^ was inserted into an AAV backbone plasmid containing the *mscL* sequence fused to the tdTomato gene and the Kir2.1 ER export signal, to drive expression at the plasma membrane. An AAV2.7m8 vector was used for intravitreous delivery. For targeting neurons in the V1 cortical layers, the SNCG promoter was replaced by the CamKII promoter and an AAV9.7m8 vector was chosen. Recombinant AAVs were produced by the plasmid co-transfection method, and the resulting lysates were purified by iodixanol purification^31^.

### US stimulus

Three focused US transducers with different central frequencies were used: 0.50 MHz (diameter, *Ø* = 1.00″ = 25.4 mm; focal distance, *f* = 1.25″ = 31.7 mm) (V301-SU, Olympus), 2.25 MHz (*Ø* = 0.50″ = 12.7 mm, *f* = 1.00″ = 25.4 mm) (V306-SU, Olympus) and 15.00 MHz (*Ø* = 0.50″ = 12.7 mm, *f* = 1.00″ = 25.4 mm) (V319-SU, Olympus), corresponding to numerical apertures of *F*/*Ø* = 1.25 and 2.00. Acoustic fields radiated by those three focused transducers are presented in Fig. 1 (simulations) and Extended Data Fig. 3 (experimental measurements). A TiePie Handyscope (HS5, TiePie Engineering) was used to produce the stimulus waveform, which was then passed through an 80 dB RF power amplifier (VBA 230-80, Vectawave) connected to the transducer. Transducer pressure outputs (pressure at focus, three-dimensional (3D) pressure maps) were measured in a degassed water tank with a Royer–Dieulesaint heterodyne interferometer^47^. US stimuli used for ex vivo and in vivo stimulation had the following characteristics: 1 kHz pulse repetition frequency with a 50% duty cycle, sonication duration between 10 and 200 ms and interstimulus interval between 0.01 and 2.00 s. Peak acoustic pressures ranged from 0.11 to 0.88 MPa, 0.30 to 1.60 MPa and 0.20 to 1.27 MPa for the 0.50, 2.25 and 15.00 MHz transducers, respectively. The corresponding estimated spatial peak pulse average intensity (Isppa) values were 0.39–25.14, 2.92–83.12 and 1.30–52.37 W cm^–2^.

### Intravitreous gene delivery and retinal imaging

Rats were anaesthetized^48^ and AAV suspension (2 µl), containing between 8 and 14 × 10^10^ viral particles, was injected into the centre of the vitreous cavity. One month later, tdTomato fluorescence imaging was performed on the injected eyes, with a MICRON IV retinal imaging microscope (Phoenix Research Laboratories) and Micron Discover v.2.2.

### MEA recordings

Retinal pieces were flattened on a filter membrane (Whatman, GE Healthcare Life Sciences) and placed on an MEA (electrode diameter, 30 µm; spacing, 200 µm; MEA256 200/30 iR-ITO, MultiChannel Systems) coated with poly-l-lysine (0.1%, Sigma), with RGCs facing the electrodes^31^. AMPA/kainate glutamate receptor antagonist 6-cyano-7-nitroquinoxaline-2,3-dione (CNQX, 25 μM, Sigma-Aldrich), the NMDA glutamate receptor antagonist [3H]3-(2-carboxypiperazin-4-yl) propyl-1-phosphonic acid (CPP, 10 μM, Sigma-Aldrich) and a selective group III metabotropic glutamate receptor agonist, l-(+)-2-amino-4-phosphonobutyric acid (LAP4, 50 μM, Tocris Bioscience), were bath applied through the perfusion line. Light stimuli were delivered with a digital micromirror display (Vialux; resolution, 1,024 × 768) coupled to a white light-emitting diode light source (MNWHL4, Thorlabs) focused on the photoreceptor plane (irradiance, 1 µW cm^–2^). US transducers were coupled with a custom-made coupling cone filled with degassed water and mounted on a motorized stage (PT3/M-Z8, Thorlabs) placed orthogonally above the retina. The reflected signal of the MEA chip and the retina was detected with a US key device (Lecoeur Electronique). The distance between the retina and transducer was equal to the focal length of the transducer; this was verified with the flight time of the reflected signal. From RGC recordings with a 252 channel preamplifier and MC_Rack v. 4.6.2 (MultiChannel Systems), spikes were sorted with Spyking CIRCUS 0.5 software^49^. RGC responses were analysed with custom scripts written in MATLAB (MathWorks 2018b) for classification as ON, ON–OFF or OFF, with the response dominance index^50^. Latencies were calculated as the time between stimulus onset and the maximum of the derivative of the spike density function (SDF). Two classes of US-responding cells were identified on the basis of latency—SL and LL—by fixing a threshold equal to the minimum of the latency distribution of the responses of NT cells to US (45 ms). We determined the peak value *A* of the SDF for the calculation of response duration, which was defined as the time interval between the two time points for which the SDF was equal to *A*/*e* (where *A* is peak depolarization and *e* is Euler’s number). The Fano factor, quantifying spike count variability, was calculated as the ratio of the variance of the spike count to the mean. The Euclidean distance between two activated cells was weighted according to the maximum firing rate of the cells. The ratio of the number of activated cells to the size of the area stimulated on the MEA chip was calculated considering the size of the US focal spot for 2.25 and 15.00 MHz and the size of the MEA for 0.50 MHz, because the focal spot was larger than the MEA for this frequency. The centre of the response was estimated by weighting the maximum firing rate of each cell by its distance from other responding cells, and the displacement of the response was calculated as the Euclidean distance between two centre-of-response positions.

### Intracranial injections

AAV suspensions were injected into the right hemisphere at two different locations in rats (2.6 mm ML, 6.8 mm AP and 3.1 mm ML, 7.2 mm AP from the bregma) or at one location in mice (2.5 mm ML, 3.5 mm AP from the bregma)^48^. For rat injections, the suspension (200 nl containing 0.2–8.0 × 10^15^ viral particles) was injected at three different depths (1,100, 1,350 and 1,500 µm from the cortical surface) with a microsyringe pump controller (Micro4, World Precision Instruments) operating at a rate of 50 nl min^–1^ and 10 µl Hamilton syringe. In mice, the AAV suspension (1 µl containing 0.2–8.0 × 10^15^ viral particles) was injected at 400 µm from the cortical surface at a rate of 100 nl min^–1^.

### In vivo extracellular recordings

One month after AAV injections, a small craniotomy (5 × 5 mm^2^) was performed above V1 in the right hemisphere^48^. The tdTomato fluorescence was checked with a MICRON IV retinal imaging microscope and Micron Discover v. 2.2 (Phoenix Research Laboratories). A 32 site µEcog electrode array (electrode diameter, 30 µm; electrode spacing, 300 µm; FlexMEA36, MultiChannel Systems) was positioned over the transfected region or in a similar zone for control rats. MEA recordings were performed with a 16 site silicon microprobe tilted at 45° to the brain surface (electrode diameter, 30 µm; spacing, 50 µm; A1x16-5mm-50-703, NeuroNexus Technologies) and MC_Rack v. 4.6.2. The MEA was advanced 1,100 µm into the cortex with a three-axis micromanipulator (Sutter Instruments). US transducers were coupled to the brain with a custom-made coupling cone filled with degassed water and US gel on a motorized stage. The distance between the cortex and transducer was equal to the focal length of the transducer. Visual stimuli were generated by a white-light-collimated light-emitting diode (MNWHL4, Thorlabs) placed 15 cm away from the eye (4.5 mW cm^–2^ at the cornea). Recordings were digitized with 32 channel and 16 channel amplifiers (model ME32/16-FAI-μPA, MultiChannel Systems). The µEcog recordings were analysed with custom-developed MATLAB scripts and the MEA recordings were analysed with Spyking CIRCUS software and custom-developed MATLAB scripts. The response duration was calculated as the interval between the two time points at which the cortical-evoked potential was equal to *A*/*e*. The activated area was defined as the area of the pseudocolour activation map over which peak depolarization exceeded the background-noise level calculated as 2 × s.d. of the signal. The response centre was estimated by weighting the peak depolarization of each electrode by its distance from the other electrodes. Its relative displacement when moving the US transducer was calculated as the Euclidean distance of the two positions. For intracortical recordings, cell latency was estimated as the time between stimulus onset and the maximum of the derivative of SDF.

### Surgery for in vivo behavioural testing

C57BL6J mice were subcutaneously injected with buprenorphine (0.05 mg kg^–1^) (Buprécare, Axience), and dexamethasone (0.7 mg kg^–1^) (Dexazone, Virbac). Animals were anaesthetized with isoflurane (5% induction and 2% maintenance, in an air/oxygen mixture) and the head was shaved and cleaned with an antiseptic solution. Animals were head fixed on a stereotactic frame with an isoflurane delivery system and eye ointment, and a black tissue was applied over the eyes. The body temperature was maintained at 37 °C. After a local injection of lidocaïne (4 mg kg^–1^) (Laocaïne, Centravet), an incision was made on the skin. Two screws were fixed in the skull, after a small craniotomy (approximately 5.0 × 5.0 mm^2^) was performed above V1 in the right hemisphere (0.5 mm steel drill) and a cortex buffer was applied. The cortex was covered with a TPX plastic sheet (125 µm thick) and sealed with dental acrylic cement (Tetric Evoflow). For behavioural experiments, a metallic headbar (PhenoSys) for head fixation was then glued to the skull on the left hemisphere with dental cement (FujiCEM 2). Animals were placed in a recovery chamber, with a subcutaneous injection of physiological serum and ointment on the eyes (Ophtalon, Centravet). Buprenorphine was injected during post-surgery monitoring.

### Mouse behavioural tests

Mice were placed on a water restriction schedule until they reached approximately 80–85% of their weight. Following habituation to the test conditions^36^, mice were trained to respond to an LS by performing a voluntary detection task: licking a waterspout (blunt 18 gauge needle, approximately 5 mm from the mouth) in response to white-light full-field stimulation (200 and 50 ms long) of the left eye (dilated with tropicamide, Mydriaticum Dispersa) over 35 trials per stimulation duration and therefore 70 trials per day. Water (~4 μl) was automatically dispensed 500 ms after the light was switched on, through a calibrated water system. The behavioural protocol and lick detection were controlled by a custom-made system^36^. The next four days (two-day break during the weekend), US stimulations were delivered on V1 for 50 ms at three different pressure values (0.2, 0.7 and 1.2 MPa). These pressure values were delivered in a different order each day (35 trials each). The intertrial intervals randomly varied and ranged between 10 and 30 s. The 15 MHz US transducer was coupled to the brain with a custom-made coupling cone filled with water and US gel. The success rate was calculated by counting the number of trials in which the mice performed anticipatory licks (between stimulus onset and the opening of the water valve). The anticipatory lick rate (Fig. 6e) for the session was calculated by subtraction from the anticipatory lick rate of a trial, the spontaneous lick rate (calculated on all the 1 s time windows before each individual stimulus onset (Fig. 6a) for all the trials) and multiplication by the success rate. Lick latency was calculated by determining the time to the first anticipatory lick after stimulus onset. The mice retained for analysis presented a success rate superior or equal to 60% on the fourth day following LS. Then, light or US sessions showing a compulsive licking behaviour were excluded based on the outlier identification made using the ROUT method (*Q* = 1%) on the session’s spontaneous lick rate averaging the measurements on all the trials of the session in the 1 s time window before the stimulus onset of the trial.

### Immunohistochemistry and confocal imaging

Samples were incubated overnight at 4 °C with a monoclonal anti-RBPMS antibody (1:500, rabbit; ABN1362, Merck Millipore) for the retina^31^, with a monoclonal anti-NeuN antibody (1:500, mouse, clone A60; MAB377, Merck Millipore) for brain sections^48^. The sections were then incubated with secondary antibodies conjugated with Alexa Fluor 488 (1:500, donkey anti-mouse and donkey anti-rabbit IgG 488, polyclonal; A-21202 and A-21206, Invitrogen, respectively) and DAPI (1:1,000; D9542, Merck Millipore) for 1 h at room temperature. An Olympus FV1000 confocal microscope with ×20 objective (UPLSAPO 20XO with a numerical aperture of 0.85) was used to acquire the images of flat-mounted retinas and brain sections (FV10-ASW v. 4.2 software).

On the confocal images processed with Fiji (ImageJ v. 1.53q), RBPMS- and NeuN-positive cells were automatically counted with the ‘analyze particles’ plugin. The cells were manually counted by two different users, with the ‘cell counter’ plugin. Quantification was performed by acquiring confocal stacks in at least four randomly chosen transfected regions of 0.4 mm^2^ (Extended Data Fig. 1). For V1 neurons, the sagittal brain slice with the largest tdTomato fluorescence zone was selected for each animal. A region of interest was manually defined in V1 and the quantifications were performed in at least six randomly chosen regions of 0.4 mm^2^.

### US-induced tissue-heating simulations

A three-fold process was used for the estimation of thermal effects: (1) simulation of the acoustic fields generated by the three transducers, with realistic acoustic parameters; (2) verification that nonlinear acoustics did not play an important role in heat transfer; and (3) realistic simulations of heat transfer and temperature rise induced at the focus by US in a linear regime for the parameters used in this study.

For nonlinear simulations, we used MATLAB’s k-Wave toolbox by defining the geometry of the transducer in three dimensions and using the following parameters for the propagation medium (water): sound speed, *c* = 1,500 m s^–1^; volumetric mass, *ρ* = 1,000 kg m^–3^; nonlinearity coefficient, *B*/*A* = 5; attenuation coefficient, *α* = 2.2 × 10^–3^ dB cm^–1^ MHz^–*y*^; frequency power law of the attenuation coefficient, *y* = 2 (ref. ^51^). We simulated quasi-monochromatic 3D wavefields using long bursts of 50 cycles; this gave us the maximum pressure field in three dimensions as well as the waveform at the focus. Simulations were calibrated by adjusting the input pressure (excitation of the simulated transducer) to reach the pressure at the focus measured in the water tank with real transducers. The full-width at half-maximum (FWHM) focal-spot diameter in the *x*–*y* plane was 4.360, 1.610 and 0.276 mm, and the length of the major axis in the *x*–*z* plane was 32.3, 20.6 and 3.75 mm for the 0.50, 2.25 and 15.00 MHz transducers, respectively (Fig. 1b–d). Nonlinear effects were evaluated by estimating the relative harmonic content of the waveform at the focus. In the 15 MHz focus transducer example in Fig. 1d, the experimental and simulated signals at the focal spot were compared and found to be highly concordant (Extended Data Fig. 4a). Furthermore, the amplitude of the second harmonic is 19.8 dB below the fundamental (20.9 dB in the simulated case), meaning that if the fundamental energy is *E*, the second harmonic has energy *E*/95 (Extended Data Fig. 4b). Therefore, we can reasonably neglect the nonlinear effects in the calculations of the thermal effects, as they account for ~1% of the energy involved. The same conclusions were drawn at 0.5 MHz and 15.0 MHz. Linear wave propagation approximations considerably decreased the computing cost of the simulations. Linear propagation simulations were conducted with the Field II toolbox in MATLAB^52,53^, in the monochromatic mode, with the same medium properties as k-Wave (water), to obtain the 3D maximum pressure fields. These maximum pressure fields were used to build a heating source term $Q_{\mathrm{US}} = \frac{{\alpha _{\mathrm{np}}p_{\mathrm{max}}^2}}{{\rho _\mathrm{b}c_\mathrm{b}}}$, where *α*_np_ is the absorption coefficient of the brain at the considered frequency (59.04 Np m^–1^ at 15 MHz, calculated from *α*_brain_ = 0.21 dB cm^–1^ MHz^–*y*^ and *y* = 1.18), the brain volumetric mass *ρ*_brain_ = 1,046 kg m^–^^3^, the brain sound speed *c*_brain_ = 154 s^–1^ and *p*_max_ is the 3D maximum pressure field. This source term was then used in the resolution of a Penne’s bioheat equation $\rho _{\mathrm{brain}}C_{\mathrm{brain}}\times\frac{{\partial T}}{{\partial t}} = \mathrm{div}\left( {K_\mathrm{t}\times\nabla T} \right) - \rho _{\mathrm{blood}}C_{\mathrm{blood}}P_{\mathrm{blood}}\left( {T - T_\mathrm{a}} \right) + Q$ in k-Wave, where *C*_brain_ is the blood specific heat capacity (3,630 J kg^–1^ °C^–1^), *K*_t_ is the brain thermal conductivity (0.51 W m^–1^ °C^–1^), *ρ*_blood_ is the blood density (1,050 kg m^–3^), *C*_blood_ is the blood specific heat capacity (3,617 J kg^–1^ °C^–1^), *P*_blood_ is the blood perfusion coefficient (9.7 × 10^–3^ s^–1^), *T*_a_ is the arterial temperature (37 °C), *Q* = *Q*_US_ + *ρ*_brain_*γ*_*brain*_ and *γ*_brain_ is the heat generation of the brain tissue (11.37 W kg^–1^) (refs. ^54,55^). The initial condition for brain temperature was set to *T*_0_ = 37 °C.

This simulation corresponds to the worst-case scenario regarding the given temperature rise. (1) The acoustic propagation is simulated in water only (non-derated value), with a lower attenuation coefficient (2.2 × 10^–3^ dB cm MHz^–2.00^) than the brain (0.59 dB cm MHz^–1.27^), even if a part of the propagation occurs within the brain. The *p*_max_ maps are, therefore, overestimated. (2) Thermal absorption is simulated in the brain tissue only, with a higher absorption coefficient (0.21 dB cm MHz^–1.18^) than water, even if a part of the maximum pressure field is actually located within the water of the acoustic coupling cone. Therefore, *Q*_US_ is slightly overestimated. We mapped the temperature in three spatial dimensions and time, and looked for the point of maximum temperature rise (Extended Data Fig. 4c–f).

### Statistical analysis

Statistical analyses were carried out with Prism software (Prism 9, GraphPad). Values are expressed and represented as mean values ± standard error of the mean (s.e.m.) on figures and in the text, unless specified otherwise. Data were analysed in unpaired Welch’s *t*-tests (two tailed) or an unpaired multiple *t*-test with Sidak–Bonferroni correction for multiple comparisons. Statistical tests are provided in the figure legends.

### Reporting summary

Further information on research design is available in the Nature Portfolio Reporting Summary linked to this article.

## Online content

Any methods, additional references, Nature Portfolio reporting summaries, source data, extended data, supplementary information, acknowledgements, peer review information; details of author contributions and competing interests; and statements of data and code availability are available at 10.1038/s41565-023-01359-6.

## Supplementary information

Reporting Summary

## Data Availability

Data supporting the findings of this study are available within the Article and via FigShare at https://figshare.com/projects/Ectopic_expression_of_a_mechanosensitive_channel_confers_spatiotemporal_resolution_to_ultrasound_stimulations_of_neuronal_circuits_for_visual_restoration/154041. All other data are available from the corresponding author upon reasonable request. Source data are provided with this paper.

## Source data

Source Data Fig. 1 Excel files of data points.
Source Data Fig. 2 Excel files of data points.
Source Data Fig. 3 Excel files of data points.
Source Data Fig. 4 Excel files of data points.
Source Data Fig. 5 Excel files of data points.
Source Data Fig. 6 Excel files of data points.
Source Data Extended Data Fig. 2 Excel files of data points.
Source Data Extended Data Fig. 4 Excel files of data points.
Source Data Extended Data Fig. 5 Excel files of data points.
Source Data Extended Data Fig. 6 Excel files of data points.

## Code availability

The custom MATLAB codes are available from the corresponding author upon request.
